# Profiling X chromosome genes expression relevant to sex dimorphism in stroke: insights from transcriptomics landscape analysis

**DOI:** 10.3389/fgene.2025.1479270

**Published:** 2025-03-21

**Authors:** Xiu-De Qin, Yue-Rong Li, Qian Cai, Jia-Ye Liu, Zhao-Hui Dang, Li-Ling Li, Jia-Wei Min, Shao-Hua Qi, Fan Bu

**Affiliations:** ^1^ Department of Neurology and Psychology, Shenzhen Traditional Chinese Medicine Hospital, The Fourth Clinical Medical College of Guangzhou University of Chinese Medicine, Shenzhen, Guangdong, China; ^2^ State Key Lab of Structural Chemistry, Fujian Institute of Research on the Structure of Matter, Chinese Academy of Sciences, Fuzhou, Fujian, China; ^3^ School of Public Health, Shenzhen University Medical School, Shenzhen, Guangdong, China; ^4^ College of Biomedical Engineering, South-Central Minzu University, Wuhan, Hubei, China; ^5^ Systems Medicine and Bioengineering, Houston Methodist Hospital, Houston, TX, United States

**Keywords:** differentially expressed genes, sex chromosomes, sex dimorphism, stroke, transcriptomics

## Abstract

**Introduction:**

Although age is the most important non-modifiable risk factor for cerebral stroke, it is also apparent that females commonly exhibit longer lifespan and better outcome after stroke compared to the age-matched males. A critical event after stroke is the peripheral infiltration of immune cells across damaged blood-brain barrier, which induces inflammatory and immune responses within the brain parenchyma and consequently worsen brain injury. These events are also dependent on age and display a sex different pattern. Theoretically, X chromosome-encoded differential expression genes (DEGs) may explain differences between the sexes. However, the expression and regulation of these DEGs after stroke have not been studied in detail.

**Methods:**

We conducted three datasets of human blood cells, mice brain, mice microglia and T cells that were previously published, and analyzed the contribution of gender, age and stroke insult on the X chromosome-encoded DEGs.

**Results:**

The main findings were (i) compared to age, the stroke/hypoxia was a more potent factor in eliciting the DEGs. Particularly, older stroke patients exhibited more changes compared to young stroke group. (ii) After a stroke, the DEGs was diversely influenced by sex, age and cell types being studied. Particularly, either aging or gender led to more striking changes in brain-infiltrating T cells than in the resident immune cells.

**Discussion:**

These findings highlight the complex interplay between sex, age, and immune responses in mediating stroke incidence and outcome. Investigation of the identified X chromosome-encoded genes in brain-infiltrating T cells deserves high priority, as they may play more important roles in explaining gender-related differences in stroke and brain injury.

## 1 Introduction

The 2022 Global Stroke Fact Sheet indicates that more than 12.2 million individuals suffered a stroke during that year (Feigin et al., 2022). This is equivalent to approximately one new stroke patient in every 3 s. Representing more than 85%, the incidence of ischemic strokes (IS) is rising compared to hemorrhagic strokes (HS) (Feigin et al., 2022; Owolabi et al., 2021; Benjamin et al., 2018; Howe and McCullough, 2015). Over the past decades, various risk factors have been identified, including aging, elevated systolic blood pressure, high body mass index, raised fasting glucose, air pollution, smoking, poor diet, high levels of low-density lipoprotein cholesterol, alcohol use, and reduced physical activity (Juli et al., 2022; Yoon and Bushnell, 2023; Soliman et al., 2018; Alawneh et al., 2020).

The blood-brain barrier, which normally restricts the passage of cells and molecules from the bloodstream into the brain, becomes compromised following a stroke (Nian et al., 2020; Gao et al., 2023). This breach allows peripheral immune cells, such as T cells and monocytes, to infiltrate the brain. The infiltrating cells contribute to the inflammatory response, which is a critical component worsening brain injury (Candelario-Jalil et al., 2022). Research has shown that the inflammatory response accompanied with blood-brain barrier integrity can vary significantly between males and females (Candelario-Jalil et al., 2022; Dion-Albert et al., 2022). These differences may be attributed to variations in hormone levels, gene expression and immune response. For instance, the inflammatory response is commonly more pronounced and leads to greater neuronal damage in males (Qi et al., 2021). Understanding sex-specific patterns in immune response after stroke is crucial for developing targeted therapies that can effectively address the unique needs of male and female patients.

Data from retrospective cohort studies demonstrated that young women experience better outcomes than age-matched males, and the incidence of ischemic events is higher in men throughout most of the life span (Appelros et al., 2003; Bots et al., 2017; Arnold et al., 2023; Phan et al., 2021). However, with increasing age women become more susceptible to stroke while showing poorer recovery and post-stroke quality of life due to, at least partially, the loss of estrogen after menopause (Towfighi et al., 2007; Benjamin et al., 2019; McCullough et al., 2001; Reeves et al., 2008). In addition, research to date provided more evidence that affect sex difference of stroke, including biologic, behavioral, and social factors (Roy-O’Reilly and McCullough, 2018; Bale and Epperson, 2017). For instance, sex chromosomes observably performed sexual dimorphism in mediating prognosis after ischemic stroke. Using XY^*^ mouse model, people demonstrated that both aged and young XX and XXY mice had worse stroke outcomes compared to XO and XY mice, respectively (Qi et al., 2022). In addition, X-chromosome inactivation (XCI) is known a compensatory mechanism balancing gene expression levels between the two sexes that moderates physiologic and developmental changes. Interestingly, some genes escape XCI and show higher transcript abundance in females that may explain sex-linked differences of disease susceptibility and severity, including cerebral stroke (Qi et al., 2021; Sun et al., 2022; Migeon, 2020; Posynick and Brown, 2019). Although the contribution of sex chromosomes and XCI genes was well established in stroke, omics analysis of differentially expressed genes (DEGs) is limited, and that their effect was thought to be of little direct consequence in stroke pathology. Additionally, the comparative analysis of these genes under the overlapping influence of sex, age, and cell-specific expression patterns has largely been ignored (Qi et al., 2021).

Large datasets of RNA sequencing (RNA-seq) from clinical and experimental studies identified numerous molecules regulated in response to the insult of a stroke, suggesting that these play a role in disease pathophysiology. However, few studies focused on the expression of X chromosome-encoded genes, despite the clearly documented differences between the two sexes. We herein reported our findings based on the analysis of publicly accessible transcriptomic datasets, examining the expression of X chromosome-encoded genes in human blood cells, mouse whole brain tissues and specific immune cells. Particular attention was directed at T cells, microglia, macrophages, and neutrophils, in the hope of identifying key features for explaining the dichotomy of stroke manifestations in the two sexes.

## 2 Methods

### 2.1 Data resources and ethics statement

The data is available from National Center for Biotechnology Information and Gene Expression Omnibus, where allowed researchers to download and reuse public datasets for scientific purposes without ethics approval. The data was filtered based on sex, age, type of stroke, and the sequencing platform used. To analyze gender differences in humans, we selected the transcriptomic profiles from peripheral monocytes, neutrophils, and whole blood from 38 IS patients and 18 control individuals from the dataset published by Carmona-Mora (Carmona-Mora et al., 2023). To analyze differences in mice, we used the GSE137482 dataset, that included 3- and 18-months old C57BL/6 mice subjected to permanent middle cerebral artery occlusion to model cerebral ischemia (N = 6/group) (Androvic et al., 2020). In addition, the GSE174574 single-cell RNA-seq data published by Zheng was reanalyzed (N = 3/group) (Zheng et al., 2022). Gene expression data from the GSE137482 and GSE174574 datasets were downloaded for validation purposes. The data was imported into R (version 4.0.2) for downstream analysis. RStudio (2022.07.1) was used for statistical analyses and to create image plots. Sample information and the allocation of figures is summarized in Supplementary Table S1.

### 2.2 Analysis of gene expression and enrichment

The analysis of gene expression was performed in R and RStudio. DEGs analysis was carried out using the DESeq2 algorithm (v 1.32.0) (Love et al., 2014; Zhu et al., 2018). For the enrichment analysis of DEGs transcripts, we utilized the ClusterProfile package (v 3.18.1) (Wu et al., 2021; Yu et al., 2012) accepting a *p*-adjusted value <0.05 as the cutoff for biologically relevant enrichment results. All analyses were conducted in RStudio, with the results being plotted using ggplot2.

### 2.3 Statistical analyses

Data were presented as mean ± S.D. Difference was assessed by multiple t-test for two individual groups, and by two-way ANOVA with Tukey post hoc tests among multiple groups (GraphPad Prism Software Inc., San Diego, CA). In order to control the confounding variables, a multiple regression was conducted in analyzing human data, in which the dependent variable was gene expression variable, while independent variables were the risk factors affecting stroke incidence and outcome, including gender, age, diabetes, hypercholesterolemia and hypertension. R (version 4.0.2) was used for statistical analyses and for generating image plots. Statistical significance was defined as *p* < 0.05.

## 3 Results

### 3.1 Dichotomy of stroke incidence between sexes over time

**FIGURE 1:**
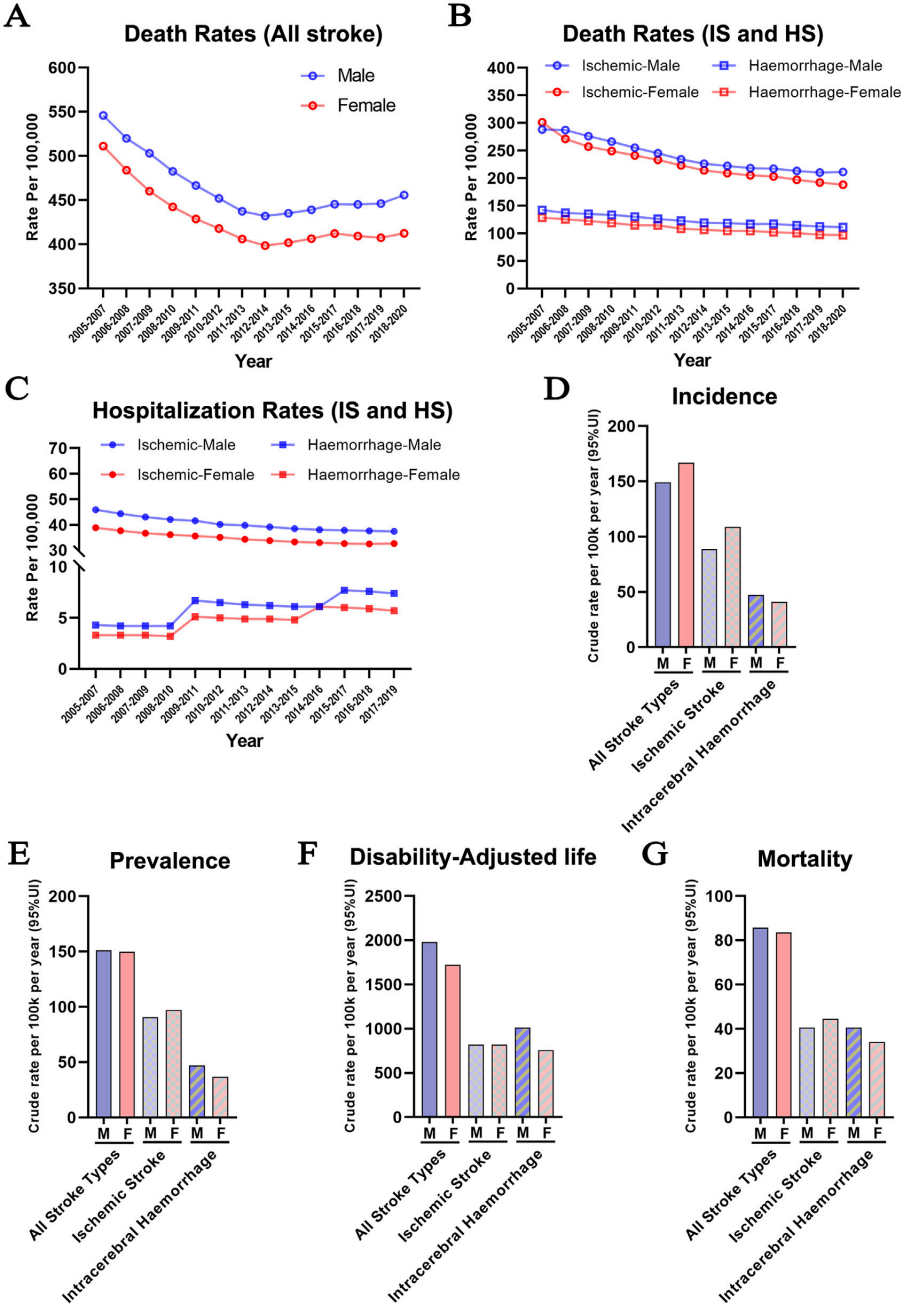
Dichotomy of the incidence of ischemic and hemorrhage stroke between the two sexes **(A)** Stroke mortality rates in males and females in the U.S. population between 2005 and 2020 (All Races/Ethnicities, >35 years old) **(B)** The distribution of ischemic and hemorrhagic stroke mortality rates in males and females between 2005–2020 (All Races/Ethnicities, >35 years old) **(C)** Hospitalization rate due to ischemic and hemorrhage stroke in males and females between 2005–2019 (All Races/Ethnicities, >35 years old) (https://nccd.cdc.gov/DHDSPAtlas/?). **(D–G)** Aged-adjusted incidence **(D)**, prevalence **(E)**, disability-adjusted life years **(F)**, and mortality **(G)** per 100,000 people for both ischemic and hemorrhagic stroke in men and women (World Stroke Organization: Global Stroke Fact Sheet 2022).

To track the long-term impact of sex on trends of stroke incidence worldwide, we analyzed new case statistics, mortality, and hospitalization rates of stroke according to the patients’ sex and type of stroke (Data source: https://nccd.cdc.gov/DHDSPAtlas/?). Between 2005 and 2020, mortality rates from all strokes, including cerebral ischemia and hemorrhage, declined from 500 to 550 to 400–450 per 100,000 population. Invariably, males have a higher mortality rate compared to females regardless of age, race, and ethnicity (Figure 1A). Previous studies largely agreed that IS was 10 times more common than HS in Western countries and carried a lower mortality risk (Namale et al., 2020; Andersen et al., 2009), although contradictory data was also reported (Abdu et al., 2021). We analyzed the mortality between 2005 and 2020 in the US (Figure 1B) and found that the likelihood of death was two-fold higher after IS during this period and this difference was not influenced by the sex of the patients. However, there was a trend toward a continuously declining mortality for all forms of stroke, reflecting the improved post-stroke management and treatment strategies.

Hospitalization rate is generally accepted to reflect the early detection and prompt treatment of stroke. However, it also provides a picture of stroke incidence. As shown in Figure 1C, the hospitalization rate for IS declined slightly between 2005 and 2019. Conversely, the hospitalization rate of HS showed an upward trend in both sexes during the same period. The underlying reasons are complex, including age-related hypertension, cerebral small vessel disease, changes in lifestyle, improved diagnostic techniques, and enhanced public awareness. In addition, females had a higher incidence of strokes, especially IS (Figure 1D). Furthermore, differences between males and females in stroke prevalence, disability-adjusted life years, and mortality show similar trends worldwide (Feigin et al., 2022), with higher rates of IS in females, and a higher incidence of HS in males (Figures 1E–G).

### 3.2 Expression pattern of XCI escape genes in response to ischemic stroke in human blood samples

**FIGURE 2:**
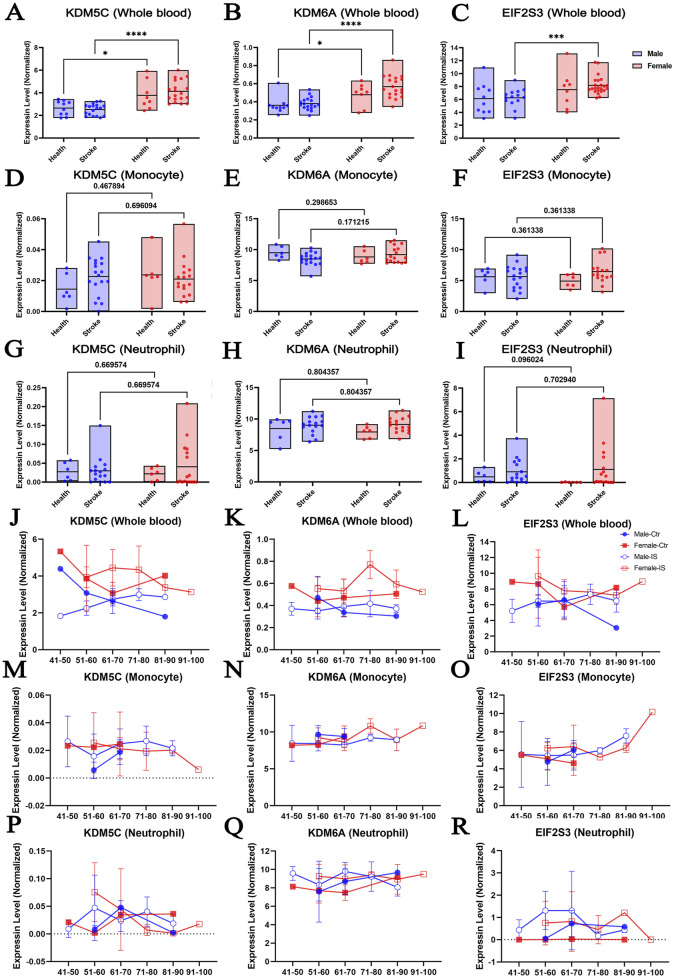
The expression of escaping XCI genes shows sexual dimorphism in response to ischemic stroke in human blood samples. The abundance of the KDM5C, KDM6A, and EIF2S3 in human whole blood **(A–C)**, monocytes **(D–F)**, and neutrophil **(G–I)** according to sex after stroke. Changes in the expression of KDM5C, KDM6A, and EIF2S3 according to age in whole blood samples **(J–L)**, monocytes **(M–O)**, and neutrophils **(P–R)** of male and female IS patients (https://bmcmedicine.biomedcentral.com/articles/10.1186/s12916-023-02766-1/tables/1). N = 6–29/group. Data was presented as mean ± S.D. Statistical significance was defined as *p* < 0.05 that was adjusted for two-way ANOVA with Tukey post hoc tests, and multivariate linear regression.

To explore if there was a difference in the expression of XCI escape genes between the sexes, we reanalyzed the transcriptomic profiles of human peripheral whole blood, monocytes and neutrophils in the RNA-seq dataset published by Carmona-Mora (Carmona-Mora et al., 2023). We focused on 19 genes that have been implicated in the onset and maintenance of XCI, including KDM5C, KDM6A, EIF2S3, ATRX, CNKSR2, DDX3X, FMR1, HDAC8, MECP2, MID1, MORF4L2, MSL3, MAGEC1, OGT, PHF6, SMC1A, SYP, USP9X, and TMEM47 (Supplementary Figures S1, S2). The datasets were already filtered and normalized by the original authors (TPM normalized, non-log, filtered features with maximum ≤40 reads were excluded). We herein explored whether stroke event could affect the sexual dimorphism expression of these 19 X chromosome-escaping genes. We found that, compared to males, females performed significantly higher expression of KDM5C and KDM6A in whole blood of healthy cohort (Figures 2A, B). This difference seemed further exacerbated by ischemic stroke, which was evidenced by that more significant differences were observed between sexes. Interestingly, EIF2S3 level was comparable between sexes in the whole blood of healthy cohort, but become significantly higher in females after stroke (Figure 2C). Importantly, a multiple regression analysis indicated that other risk factors of stroke, including age, diabetes, hypercholesterolemia and hypertension, were not associated with the sex difference of EIF2S3 level (Supplementary Table S2). It is known that circulating monocytes and neutrophils infiltrate the injured brain and mediate the inflammatory process that develops after stroke (Carmona-Mora et al., 2021; Shichita et al., 2012; Planas, 2018a). However, the expression levels of KDM5C, KDM6A and EIF2S3 were comparable between sexes with or without ischemic stress (Figures 2D–I).

It was previously reported that gene expression profiles in specific cell types change with increasing age (French et al., 2017; Chow et al., 2012; Brinkmeyer-Langford et al., 2016). Therefore, we explored whether KDM5C, KDM6A, or EIF2S3 displayed age-dependent expression changes in males and females and whether such age-dependent expression changes could be altered by ischemic stroke (Figures 2J–R). We found that the expression of KDM5C and KDM6A showed relatively smooth fluctuations in the whole blood, monocytes, and neutrophils. However, EIF2S3 expression showed a trend of increase in the monocytes of the 91–100-year-old cohort (Figure 2O). In neutrophils, IS caused an increased trend of EIF2S3 at most age groups, irrespective of the sex of the patient (Figure 2R). The level of EIF2S3 mRNA in female neutrophil is very close to 0 throughout ages, although it is clearly detectable in female brain and liver (Xu et al., 2006), suggesting that the tissue- and cell type-preferential expression of EIF2S3 might be sexual dimorphic. It is worthy to note that this observation is lack of statistical support as the sample size is insufficient in separated age bracket. More data is required in the future.

### 3.3 The impact of cerebral ischemia on the expression of X chromosome genes in mouse brains

**FIGURE 3:**
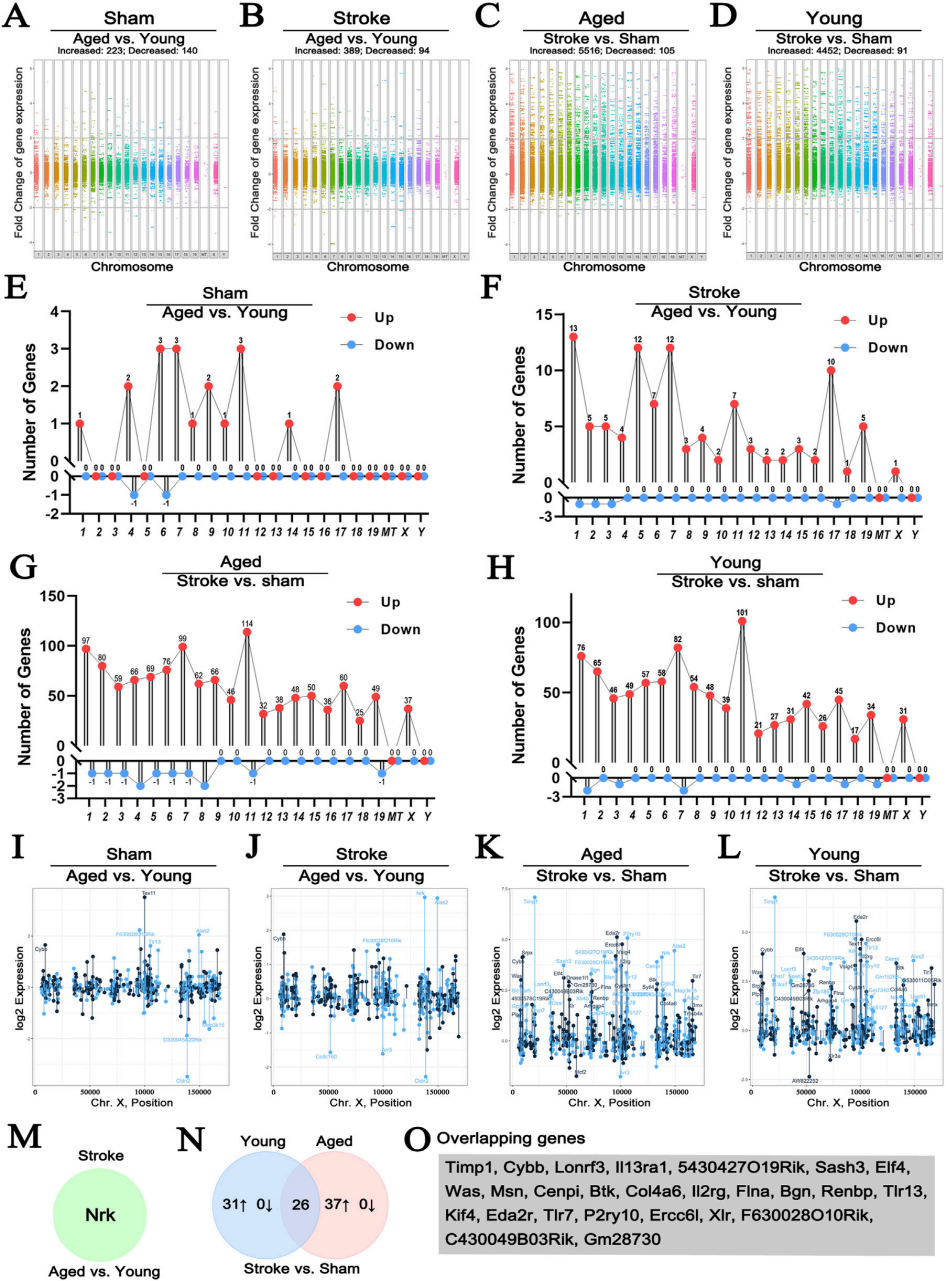
The effect of stroke and age on the expression of genes localizing to the X chromosome. Manhattan plots comparing the effect of age on genome-wide gene expression changes in sham operated or hypoxic brains **(A, B)**. Comparison of genome-wide changes in either young or old animals induced by hypoxia **(C, D)** satisfying the *p* < 0.05 and fold change>2 criteria. Young animals were 3-month-old, old ones were 18-month-old. Lollipop plots map the most profoundly altered DEGs to individual chromosomes **(E–H)**. Changes in the expression of DEGs located to the X chromosome using the same comparison strategy are shown in **(I–L)**, where each point represents the expression level of an individual sample with the indicated gene name on the X chromosome. Colors indicate “sense” strand and “antisense” strands on the double helix. Venn diagram highlighting the single gene, Nrk, that was selectively altered between old and young hypoxic brains **(M)**. The Venn diagram comparing the hypoxia-induced genes in young and old animals is shown in **(N)**, illustrating the relationship between the 31 genes upregulated in young and 37 upregulated genes in old animals. The 26 DEGs showing similar regulation patterns are listed in **(O)** (GSE137482), N = 6/group. 1–19: Chromosome 1–19, X: Chromosome X, Y: Chromosome Y, MT: Mitochondria.

To explore X chromosome-encoded DEGs according to sex and cerebral ischemia, we reanalyzed the dataset published by Androvic (GSE137482) (Androvic et al., 2020). This was derived by a bulk RNA-seq analysis of four groups, including young (3-month-old) and old (18-month-old) mice suffered sham operation and ischemia. We respectively investigated the aging effect to genes alternation under either sham (Figure 3A) or stroke (Figure 3B) condition, and that the effect of stroke insult to genes alternation under either aged (Figure 3C) or young (Figure 3D) cohort. We found that accepting a fold change of >2 with a *p* < 0.05 statistical cut off, aging led to 223 DEGs upregulated and 140 downregulated in sham animals (Figure 3A), and that aging led to 389 DEGs upregulated and 94 downregulated in stroke animals (Figure 3B). In the aged population, stroke insult led to 5,516 DEGs upregulated and 105 downregulated (Figure 3C), whereas 4,452 DEGs upregulated and 91 downregulated in young population (Figure 3D). In addition, we explored the locations of the most prominently up- or downregulated DEGs in individual chromosome. As shown in Figures 3E, F, changes associated with aging were distributed relatively evenly along autosomal chromosomes, with hardly any DEGs localizing to the X chromosome. In contrast, stroke insult led to a marked increase of DEGs on the X chromosome regardless age (Figures 3G, H).

Next, we explored DEGs localizing to the X chromosome in more detail (Figures 3I–L; Table 1). When we compared sham operated old and young animals, none of the DEGs were encoded on the X chromosome (Figure 3I), while only one gene, Nrk, significantly increased in aged stroke animals compared to young stroke group (Figures 3J, M). These findings are in line with the analysis presented above (Figures 3E, F). It is worth noting that the amplitude of expression changes was higher for genes located on the X chromosome, with −2.5 to +7.5 folds changes in both young and ages. In contrast, age alone caused −3 to +3 folds change regardless stroke insult (Figures 3K, L). Importantly, in the aged population, stroke induced 37 X chromosome-encoded DEGs upregulated, whereas stroke only induced 31 DEGs upregulated in the young population (Table 1), in which 26 DEGs overlapped with that in the aged cohort indicated by Venn diagram (Figures 3N, O). These data collectively suggest that stroke is a more robust factor affecting gene regulation than age, and this is particularly true for DEGs located on the X chromosome of mice.

**TABLE 1:** List of DEGs located to the X Chromosome in the indicated pairwise comparisons.

DEGs	Sham Aged vs Young	Stroke Aged vs Young	Aged Stroke vs Sham	Young Stroke vs Sham
Increased	0	Nrk	Timp1, Cybb, Lonrf3, Il13ra1, Dnase1l1, 5430427O19Rik, Alas2, Sash3, Elf4, Was, Slc38a5, Msn, Sytl4, Cenpi, Btk, Col4a6, Il2rg, Flna, Bgn, Renbp, Tlr13, Kif4, Eda2r, D330045A20Rik, Vsig4, Tlr7, P2ry10, Ercc6l, Cysltr1, Nrk, A630033H20Rik, Xlr, Xlr4b, F630028O10Rik, C430049B03Rik, Gm28730, Srpx	Timp1, Tex11, Cybb, Lonrf3, Il13ra1, 5430427O19Rik, Sash3, Elf4, Plp2, Was, Msn, Cenpi, Btk, Col4a6, Il2rg, Flna, Bgn, Renbp, Tlr13, Kif4, Eda2r, Chst7, Tlr7, P2ry10, Ercc6l, Xlr, G530011O06Rik, F630028O10Rik, Gm15268, C430049B03Rik, Gm28730
Decreased	0	0	0	0

Data source: GSE137482. The 26 DEGs, showing similar regulation in old and young animals are highlighted in red.

### 3.4 Sex differences of X chromosome gene expression in response to ischemic stroke in aged mice brain

**FIGURE 4:**
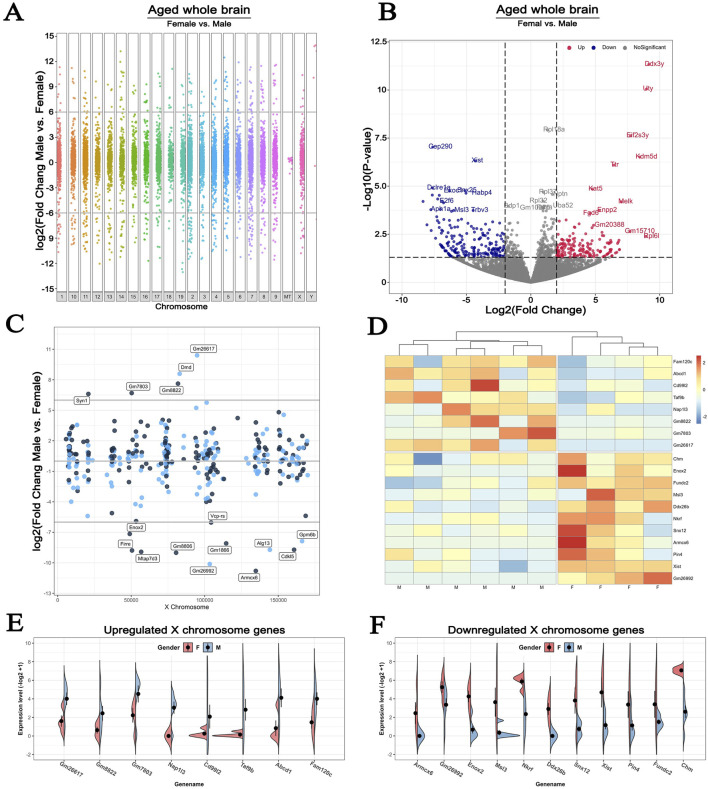
Sex differences in the expression of X chromosome-encoded genes in response to experimental hypoxia **(A)** Manhattan plot for female vs male in old mice whole brains, showing the increased and decreased genes distributed across chromosomes **(B)** The volcano plot compares DEGs localized to the X chromosome in the brains of 36-month-old female and male mice. Cutoff: fold change>2, *p* < 0.05 **(C)** Lollipop plot comparing the number of DEGs that map to the X chromosome in the whole brain of the same animals **(D)** Heatmap plot showing the up- and downregulated X chromosome-encoded DEGs with more than 6-fold difference in expression levels **(E–F)** Expression patterns of specific X chromosome-encoded genes in male and female brains. All animals in these comparisons were exposed to experimental brain hypoxia via the surgical occlusion of the middle cerebral artery (GSE137482), N = 6/group. 1–19: Chromosome 1–19, X: Chromosome X, Y: Chromosome Y, MT: Mitochondria.

Given that age and sex are major non-modifiable risk factors for stroke (Egorova et al., 2019; Curb et al., 1996), we compared the sex differences of genome-wide DEGs using the brains of 36-monthes male and female mice exposed to experimental hypoxia. In this analysis, the GSE137482 RNA-seq dataset was carried out, and that the significant difference was considered by a fold change of >6 with *p* < 0.05 statistical cut off (Figure 4A). We found that 392 transcripts were sexual dimorphic on X chromosome identified by volcano plot, in which 185 were upregulated and 207 were downregulated (fold change>2, *p* < 0.05, Figure 4B). To conduct a more accurate view of the chromosomal localization of the affected genes, we calculated the average gene expression levels within 1 kb windows tiled across the X chromosome. This analysis showed that the localization of DEGs on X chromosome was fairly evenly distributed (Figure 4C). Next, we analyzed up- and downregulated genes in male and female ischemic brains using a heatmap showing DEGs with more than 6-fold change. The upregulated X chromosome genes in males included Fam120c, Abcd1, Cd99l2, Taf9b, Nap1l3, Gm8822, Gm7803, and Gm26617; those in females were Chm, Enox2, Fundc2, Msl3, Ddx26b, Nkrf, Snx12, Armcx6, Pin4, Xist, and Gm26992 (Figure 4D). The individual variations of these DEGs in male and female brains were analyzed and visually presented in Figures 4E, F.

### 3.5 Microglial and T cells displayed different changes of X chromosome gene expression in response to stroke

It has been documented that, after a cerebral stroke, some immune cells start to infiltrate the brain and, together with brain resident immune cells, primarily the microglia, contribute to the development of brain injury (Wang H. et al., 2023; Al et al., 2019). Microglia are generally considered as “first responders” after hypoxia showing divergent features between the two sexes (Ngwa et al., 2021; Ngwa et al., 2022; Ugidos et al., 2022; Han et al., 2021a). Therefore, we analyzed the transcriptomic datasets of microglial and peripheral infiltrating T cells, to explore the expression of X chromosome-encoded DEGs in 36-months-old mouse brains after cerebral ischemia.

**FIGURE 5:**
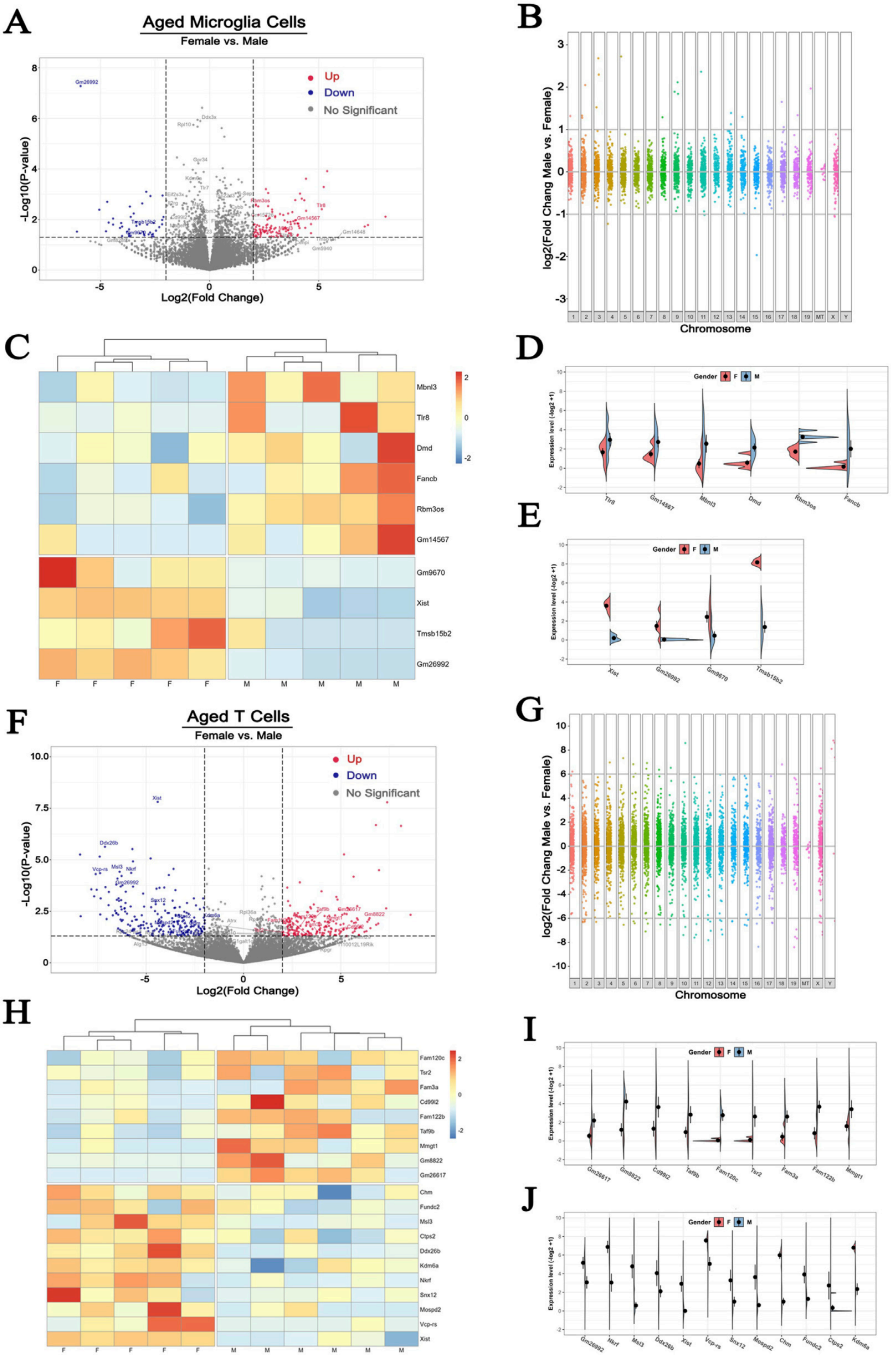
Sex differences of X chromosome-encoded gene expression responses to hypoxia in microglia and T cells **(A, F)** The volcano plots comparing DEGs in microglia and T Cells in the two sexes. Cutoff: fold change>2, *p* < 0.05 **(B, G)** Manhattan plots illustrating the difference in the distribution of DEGs across all chromosomes **(C, H)** Heatmap plots of up- and downregulated DEGs **(D, E, I, J)** Expression patterns of X chromosome-encoded genes in microglia and T Cells (GSE137482), N = 6/group. 1–19: Chromosome 1–19, X: Chromosome X, Y: Chromosome Y, MT: Mitochondria.

First, we performed a volcano plot analysis to examine differences between male and female microglial mRNA abundance. With a *p* < 0.05 and 2-fold change cutoff, we identified 168 DEGs. Of these 120 were upregulated and 48 were downregulated in female (Figure 5A). When looking at the chromosomal distribution of the DEGs, only a limited number of them were significantly altered with more than 2-folds change from the X chromosome (Figure 5B). Specifically, 6 genes, including Mbnl3, Tlr8, Dmd, Fancb, Rbm3os and Gm14567, were upregulated and 4 genes, including Gm9670, Xist, Tmsb15b2 and Gm26992, were downregulated in males compared to females (Figures 5C–E).

Signaling via activated microglia initiates the migration of peripheral blood T lymphocytes into the injured area as early as 24 h after the initial ischemic insult (Jander et al., 1995; Brait et al., 2012). This process has been documented to exacerbate the neuroinflammatory process and contributes to the ensuing brain damage (Zhang et al., 2021; Benakis et al., 2022). However, little is known about the sex dichotomy of T cell features after stroke. In the T cells from the ischemic brain of 36-month-old mice, we detected a total of 429 DEGs, in which 198 DEGs was increased and 231 was decreased in female compared to male cells (Figure 5F). Interestingly, the amplitude of change of DEGs in T cells was more pronounced than that seen in microglia. As shown in Figure 5G, some DEGs showed as high as 10-fold increase or decrease between sexes. Apart from the robust changes in the gene expression presenting on autosomes and the X chromosome, T cells also exhibited dramatic changes in the Y chromosome-encoded genes (Figure 5G). Among the 429 DEGs, 20 were located on X chromosome (Figure 5H). Of these, 9 were upregulated in males (Figure 5I), and 11 genes were upregulated in females (Figure 5J). These observations indicate that X chromosome-encoded DEGs performed more remarkable sexual dimorphism on activated T cells than on activated brain resident microglial cells.

### 3.6 Aging causes different changes of X chromosome gene expression in microglia and T cells

**FIGURE 6:**
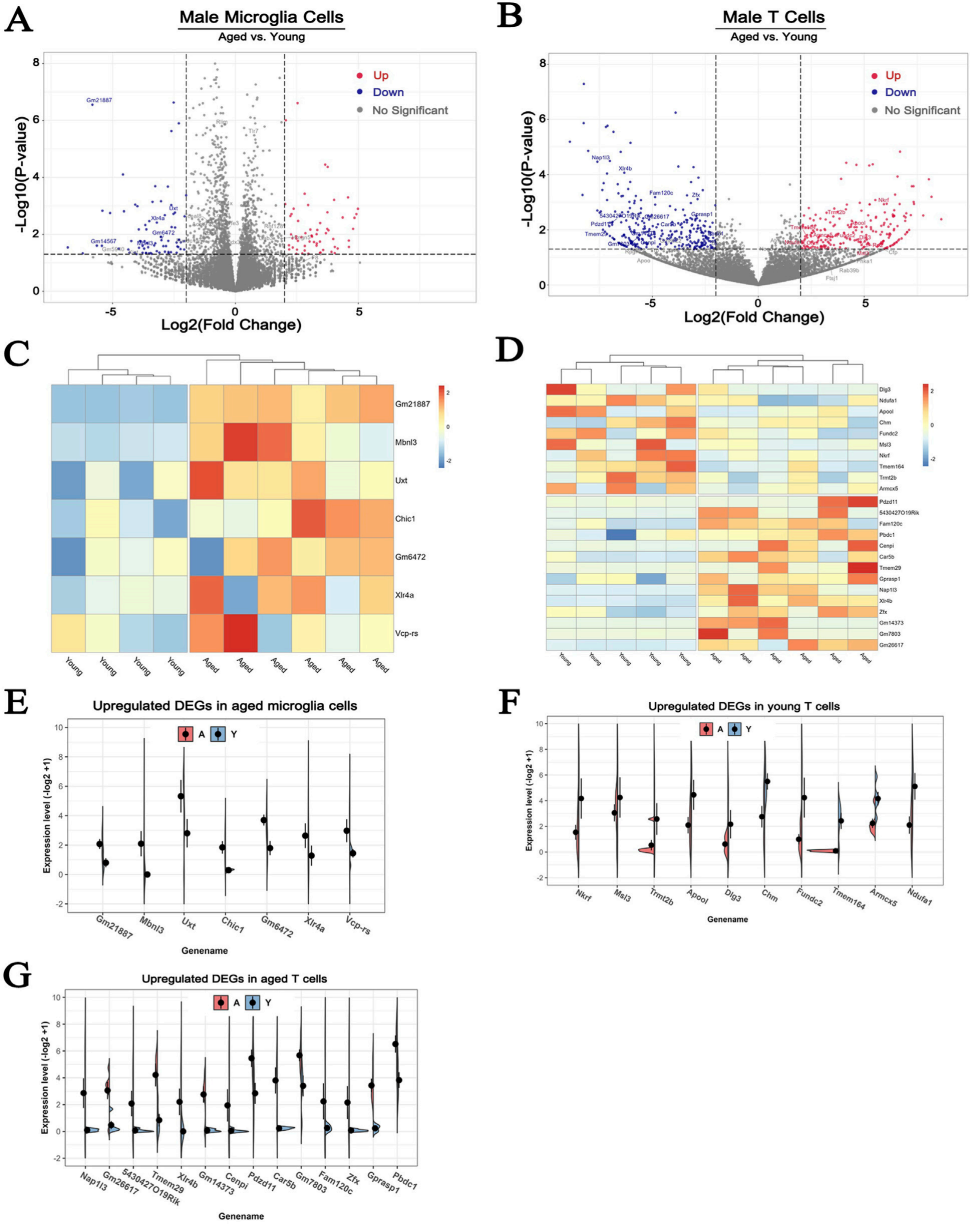
The effect of aging on the expression of X chromosome-encoded genes in microglial and T cells. **(A, B)** The volcano plots show the effect of aging on DEGs in microglial and T cells. Cutoff: fold change>2, *p* < 0.05. **(C, D)** Heatmap plot showing the up- and downregulated DEGs located on the X chromosome in microglial and T cells of male mice. **(E–G)** A more detailed comparison of the expression patterns of DEGs within the X chromosome in microglial and T cells. To avoid the confounding effect of dichotomy between the two sexes data from only male animals was used for the analyses presented here. N = 6/group.

To analyze the effect of age on hypoxia-induced changes of X chromosome-encoded DEGs on the microglial and T cells, we conducted additional comparisons to exclude the confounding effect of the differences between sexes, only data from male brains was utilized (Rebekah et al., 2022). The volcano plot analysis indicated that 61 DEGs were upregulated and 76 were downregulated in brain microglia of the 36-month-old male mice compared to the young group (Figure 6A). A similar analysis detected 229 DEGs upregulated and 290 downregulated in T cells (Figure 6B). Limiting the analysis to genes encoded on the X chromosome identified 7 upregulated DEGs in the microglia (Figure 6C), 10 upregulated and 14 downregulated DEGs in the T cells of the older animals (Figure 6D). These findings are in line with our previous observations indicating that hypoxia induces more pronounced changes of gene expression in T cells. The individual variations of these DEGs in aged microglia and T cells were visually presented in Figures 6E–G.

### 3.7 Cerebral ischemia-induced different changes of X chromosome gene expression are cell type dependent

**FIGURE 7:**
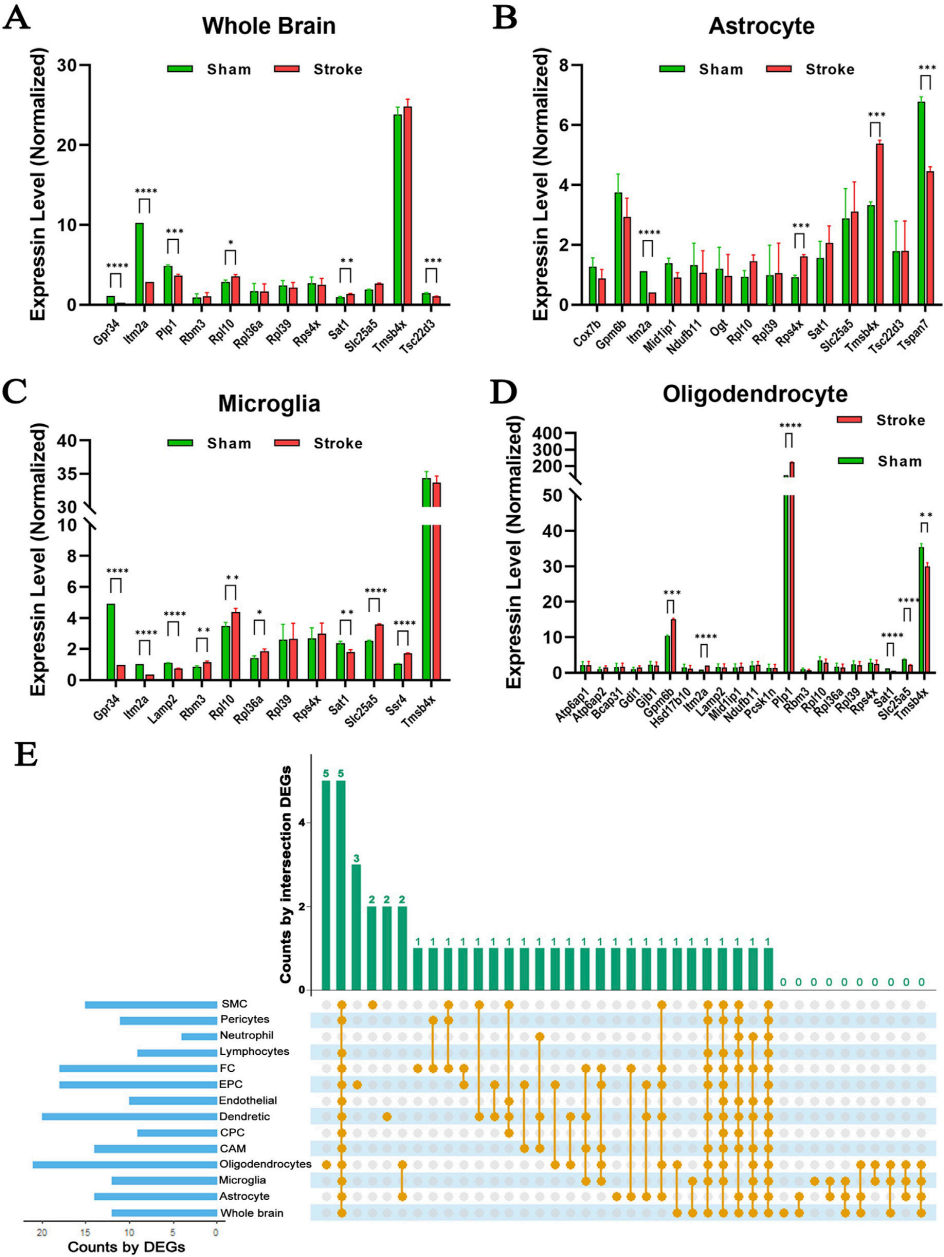
Expression profiles of X chromosome-encoded genes in different cell types in the mouse brain. **(A–D)** Gene expression pattern changes induced by hypoxia in whole brain, isolated astrocytes, microglia, and oligodendrocyte cells. **(E)** UpSet plot displaying relationships among the 14 cell types (GSE174574), N = 3/group. Data was presented as mean ± S.D. and assessed with multiple t tests, statistical significance was defined as *p* < 0.05.

A major challenge in understanding changes in hypoxia-induced gene expression patterns is the heterogeneous cellular composition of the brain. Single-cell RNA-seq is a powerful approach to gain more granular information in this context. To explore the effects of cerebral ischemia on X chromosome-encoded DEGs in individual brain cell populations, we reanalyzed the open access single-cell RNA-seq dataset (GSE174574) published by Zheng (Zheng et al., 2022). Using cell type specific gene markers, authors identified 17 principal clusters in the mouse brain and explored their transcriptional changes 24 h after permanent ischemia. We filtered and reanalyzed the expression of genes located on the X chromosome in the whole brain, astrocytes, microglia (MG), oligodendrocytes (OLG), ependymocytes (EPC), perivascular fibroblast-like cells (Fanning et al., 2019), lymphocytes (LYM), neutrophils (NEUT), pericytes (PC), venous endothelial cells (vEC), vascular smooth muscle cells (SMC), central nervous system (CNS) border-associated macrophages (CAM), choroid plexus epithelial cells (CPC), and dendritic cells (DC) (Figure 7; Supplementary Figure S2), which were also summarized (Table 2) and visualized (Supplementary Figure S3). It was notable that similar trends of gene expression were apparent across several cell types. For instance, the abundance of the transcript of Itm2a was significantly decreased in astrocytes, MG, and LYM (Figures 7B, C; Supplementary Figure S2C), but increased in OLG, EPC, PC, vEC, SMC, and CAM (Figure 7D; Supplementary Figures S2A, E, F, G., H). To summarize hypoxia-induced changes in the expression of X chromosome-encoded genes in the 14 distinct cell populations, we used an UpSet plot to indicate the detected trends (Figure 7E). These data reinforce the complexity of the expression of X chromosome-encoded genes in brain hypoxia and illustrates the challenges of understanding the differences in stroke responses between the two sexes.

**TABLE 2:** Overview of the expression pattern of X chromosome-encoded genes in various brain cell populations (GSE174574).

	Whole brain	Astrocyte	MG	OLG	EPC	FB	LYM	NEUT	PC	vEC	SMC	CAM	CPC	DC
Gpr34	↓		↓											
Itm2a	↓	↓	↓	↑	↑	−	↓		↑	↑	↑	↑	−	−
Plp1	↓			↑										−
Rbm3	−		↑	−	−	−	−		−			−		
Rpl10	↑	−	↑	−	−	−	−		−	↓	−	−	−	↓
Rpl36a	−		↑	−	−	−	−		−	−	−	−	−	↓
Gm6b				↑										
Rpl39	−	−	−	−	−	−	↓		−	−	↑	−	−	↓
Rps4x	−	↑	−	−	−	−	−		−	−	↑	−	−	↓
Sat1	↑		↓	↓		−		−	−	↓		−		−
Slc25a5	−		↑	↓	−	−	−		−	↓	−	↓	−	−
Tmsb4x	−	↑	−	↓	↓	−	↑	↑	↑	−	−	↑	↓	↓
Tsc22d3	↓					−	−	−	−	−	−	−	−	−
Ndufb11				−	−	−					−			−
Lamp2			↓	−	−	−						−		
Tspan7		↓				−								
Ssr4			↑			−						↓		−
Cybb								↑				↑		
Timp1									↓					
Msn										↓	↑		−	−
Flna											↑			
Cfp														↑

Gene expression in the whole brain, astrocytes, microglia (MG), oligodendrocytes (OLG), ependymocytes (EPC), perivascular fibroblast-like cells, lymphocytes (LYM), neutrophils (NEUT), pericytes (PC), venous endothelial cells (vEC), vascular smooth muscle cells (SMC), CNS, border-associated macrophages (CAM), choroid plexus epithelial cells (CPC), and dendritic cells (DC). ↑: upregulation; ↓: downregulation; -: no significant change; blank indicates data was unavailable. The heatmap version can be found in Supplementary Figure S3.

### 3.8 Functional enrichment characteristics of the detected X chromosome DEGs

**FIGURE 8:**
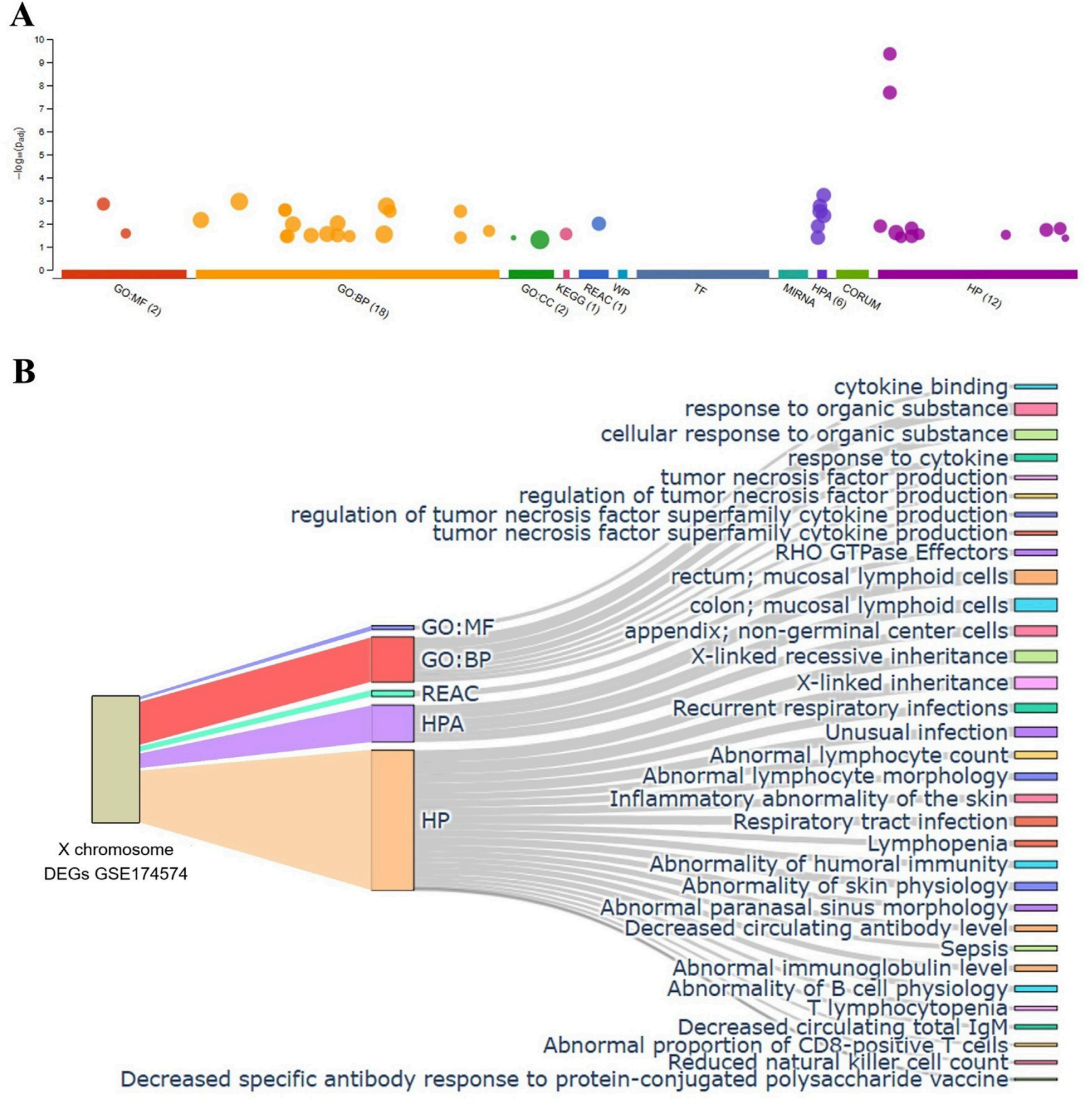
Functional classification analysis of the detected X chromosome encoded DEGs. **(A)** Graphic representation of the results analyzed by g: Profiler program (top panel, only results identified by at least three queries are shown). The most significant results for GO:MF and GO:BP pathways enrichment was shown (bottom panel). **(B)** Sankey diagram illustrating the mapping of gene set enrichment analysis based on X chromosome DEGs list of GSE174574. N = 3/group.

Next, the biologic function of the hypoxia-induced DEGs of X chromosome (refer to Figure 3) was explored by performing gene classification analyses (Dataset GSE174574). All X chromosome DEGs were uploaded into the g:Profiler (https://biit.cs.ut.ee/gprofiler/gost) functional enrichment analysis that provides known functional information sources and detects significantly enriched terms (Raudvere et al., 2019; Reimand et al., 2007). The biologic process terms were summarized in the Figure 8A. The X-axis showed the functional terms grouped by colors, in which functional enrichments were further branched. The number indicates the intersection size of query (Available in Supplementary Material).

The most relevant enriched GO terms are reported in Figure 8. Among the 26 DEGs selected (refer to Figure 3), 4 were associated with GO molecular function, cytokine binding, including IL13ra1, IL2rg, Bgn and Plp2. Notably, 18 genes associated with GO biological processes, such as response to organic substance, cellular response to organic substance, tumor necrosis factor production, regulation of tumor necrosis factor production, regulation of tumor necrosis factor superfamily cytokine production, tumor necrosis factor superfamily cytokine production, and response to cytokine. Notably, in addition to their ontology, these genes are involved in multiple terms including Reactome (REAC), Human Protein Atlas (HPA), and Human Phenotype Ontology (HP). In this enrichment analysis, stroke-induced upregulation of Toll-like receptor (TLR) 7 was associated with mucosal lymphoid cells (HPA), X-linked inheritance (HP), abnormal lymphocyte count and morphology (HP) regardless age (Available in supplementary file), which is consistent with previous report about the TLR related poor outcome and greater inflammatory response after acute ischemic stroke (Brea et al., 2011), suggesting that antagonizing TLR7 might be promising in the treatment of ischemic stroke and brain injury (Gesuete et al., 2014; Lu et al., 2020).

## 4 Discussion

It is well documented that a range of neurological diseases, including Alzheimer’s disease, Parkinson’s disease, and cerebral stroke, do not affect the two sexes equally (Gillies et al., 2014; Laws et al., 2018; Toro et al., 2019; Furr-Stimming et al., 2020; Hentosh et al., 2021; Nicoletti et al., 2023; Carcel et al., 2020). The age of women and men at the time of their first stroke is substantially different and that women normally have a poorer reported baseline function compared to men (Carcel et al., 2020; Zhang W. et al., 2023). These differences mean that sex and age should be considered when treating patients and designing clinical trials. Aging is a key risk factor for stroke as well as many other diseases, and that the aging process itself differs between males and females (Sampathkumar et al., 2020; Franceschi et al., 2018). It was proposed that the aging-related deregulation of the innate and adaptive immunity at either genetic or epigenetic levels is sex difference, which may explain diverse pathologies of certain diseases in females and males, including cerebral stroke (Taneja, 2021). Currently, there are controversial views regarding the role of sexes (X chromosome), aging, and the dichotomous biologic behavior of stroke. However, most studies that conducted expression profile analysis failed to separate these intertwined factors, and deciphered stroke pathophysiology.

The global long-term data about IS and HS has been very conspicuous, with overwhelming more males being affected mortality rate and hospitalization rate. Incidence also showed that men are more vulnerable to stroke than women. However, IS, the most common type of stroke, occurs more frequently in females. Previous microarray assays using peripheral blood samples demonstrated that IS men and women show a different expression pattern for the X chromosome-encoded genes (Stamova et al., 2012). Herein, we further determined whether the sex difference of escaping XCI could be age and cell type relevant. We selected 18 previously described genes, which have been implicated in various aspects of the onset and maintenance of XCI. Results showed that only the sex difference of EIF2S3 was profoundly affected by cerebral ischemia in whole blood, rather than in monocytes and neutrophil. Whereas age alone had no detectable effect in samples tested. Given XCI toward one parental X is skewed by sex and aging observed in multiple tissues, organs and cell types under either health or disease conditions (Zito et al., 2019; Migeon, 2007), we assume that EIF2S3 might modulate the consequence after stroke through intertwined role of mechanisms.

To explore age effect on stroke-associated gene expression patterns within the brain, we used data from a previously reported animal model. In this, young (2-month-old) and older (36-month-old) mice were subjected to either sham surgery or middle cerebral artery occlusion. A series of pairwise comparisons allowed us to differentiate between the contribution of age and hypoxia to the changes of gene expression profiles. Results clearly indicated that hypoxia had a considerably more profound effect on the expression profiles of either global or X chromosome-encoded genes than the changes by age. Focusing on the X chromosome, we found that brain hypoxia-induced dramatic alterations on the expression of genes encoded on this chromosome, whereas aging had relatively minor effects. Furthermore, changes in gene expression did not correlate with the physical location of the DEGs on the X chromosome or the “sense” or “antisense” strand of double helix. With the dataset obtained from this animal model, we can also compare differences in male vs. female RNA transcriptome at both the whole genome level and X chromosome level. This dual analysis approach allows us to identify sex-specific gene expression patterns and understand how variations on the X chromosome contribute to these differences. There were almost similar levels of up- and downregulated DEGs in the entire genome, including autosomes and the X chromosome. It is worth noting that few DEGs located on Y chromosome and no DEGs were showing more than 6-fold change on MT DNA content (Figure 4A). Comparing male and female brain tissues of old mice, we found that the upregulated X chromosome-encoded genes in males included Fam120c, Abcd1, Cd99L2, Taf9b, Nap1l3, Gm8822, Gm7803, and Gm26617 (Figure 4E). In contrast, in female brains hypoxia upregulated Chm, Enox2, Fundc2, Msl3, Ddx26b, Nkrf, Snx12, Armcx6, Pin4, Xist, and Gm26992 (Figure 4F).

Next, we investigated gene expression changes originating from X chromosome of microglia and T cells. The data showed that the alterations of gene expression patterns was more robust in T cells. It is worthy to note that histone demethylase KDM6A exhibited lower level in aged males compared to the age-matched females after ischemic stroke, which was consistently observed in either human whole blood or mice T cells. KDM6A can act as an oxygen sensor to control chromatin and cell fate (Chakraborty et al., 2019). Our previous study demonstrated that knock down KDM6A significantly suppressed the production of pro-inflammatory factors by primary microglia of either female or male mice (Qi et al., 2021). It is known that some subsets of CD4^+^ cells, such as helper T cells, play a pro-inflammatory role by activating inflammatory immune (Wang YR. et al., 2023). After stroke insult, T lymphocytes can enter the infarct region within 24 h and continue to accumulate for up to 7 weeks (Zhang et al., 2021; Selvaraj and Stowe, 2017). Infiltrated T cells exacerbate the brain lesion through facilitating thromboinflammation (Planas, 2018b). In this case, we assume that higher KDM6A of T cells might be correlated with worse prognosis in aged females, compared to aged males, because of KDM6A-drived inflammatory brain injury. In addition, within the microglial population, we found that aged males predominantly exhibited high expression of Dmd, the second largest known human gene, which is responsible for producing the dystrophin protein. It is known that Dmd mutation is one of the key reasons causing Duchenne muscular dystrophy, an X-linked recessive neuromuscular disorder, which is often associated with psychosocial abnormalities and cognitive impairment (Nowak and Davies, 2004; Zhang XF. et al., 2023). However, whether higher microglial Dmd is correlated with more optimistic cognitive recovery after stroke in aged males needs to be investigated further.

Given that the brain consists of multiple cell populations, we then analyzed single-cell RNA-seq data to detect subtle differences among these, limiting this analysis to the X chromosome only. Surprisingly, we found that the DEGs did not overlap with XCIs. While changes in the expression of several genes showed the similar trend across multiple cell types, others exhibited unique changes in response to hypoxia. For instance, Itm2a tends to be upregulated in OLG, EPC, PC, vEC, SMC, and CAM, but was downregulated in astrocytes, microglia, and LYM. In contrast, Tmsb4x was increased in astrocytes, LYM, NEUT, PC, and CAM, and decreased in EPC, CPC, and DCs. Finally, we summarized the function of the detected DEGs with functional enrichment analysis. The critical outcome of pathways including cytokine binding, response to cytokine, cytokine production, and the physiological/morphological changes of lymphocyte. A recent study indicates the lymphocyte (B cells) phenotype is intrinsically linked to beneficial or detrimental function following stroke, which has the ability to determine the healthy and disease states with particular emphasis in the context of ischemic stroke (Malone et al., 2023). In addition, people found that the post-stroke cytokines are related to the progression of brain damage. Cytokines can activate immune cells and promote tissue destruction, which in turn stimulates the expression of multiple inflammatory mediators and adhesion molecules, and consequently exacerbate brain injury (Zhu et al., 2022).

Among the X chromosome-encoded DEGs induced by ischemic stroke in both aging and young cohorts (refer to Figures 3N, O), TLR7 is worthy to be noted as it is protective against brain injury after stroke (Leung et al., 2012). Meanwhile, our functional enrichment analysis implies its roles in the sex difference of ischemic stroke (Figure 8; Supplementary Material). Mechanically, the role of TLR7 in sex differences of neurological disorders is interpreted by its function to demyelination during aging (Lopez-Lee et al., 2024). Another study used imiquimod, an agonist of TLR7, to regulate the neuroinflammatory responses of CNS and enhance the brain endothelial barrier strength (Johnson et al., 2018). Although these findings collectively support the potential of targeting TLR7 in mediating brain damage and outcome after stroke regardless sex and aging, further investigation is required in future. In the same time, our results provide preliminary, yet warrant validation, insights into the early response of ischemic stroke, such exploratory results can pave the way for future research and providing reference for mechanistic studies in the future.

Obviously, our results also need to be interpreted in the light of its potential limitations. Firstly, due to the small number of samples, we could not identify clear expression patterns of XCI genes among complex types of strokes when analyzing human blood samples. Thus, larger sample size might be required for more comprehensive conclusions. Secondly, as this study is a reanalysis of open datasets, we do not possess original data to reproduce our findings, and our conclusions are highly dependent on the quality and comprehensiveness of the available datasets. Performing an updated analysis in the future, when more robust and integrated data becomes available, would be highly valuable. Thirdly, this analysis did not consider the gene expression profiles dependent on spatial distribution, and did not thoroughly address the complex influence of confounding factors in human patients, such as age, stroke type, time window, stroke severity, and associated comorbidities because of less data available. These limitations highlight the need for further research incorporating these critical variables to enhance the reliability and applicability of the findings. Han’s recent study showed that same type of cell performed diverse spatial characteristics of gene expression in the ipsilateral hemisphere of mice after ischemic stroke (Han et al., 2021b), which raises the possibility that the expression changes of X-chromosome encoded genes might be linked to specific histologic entities with the consideration of infarct extension, infarct regions and affected artery. However, further analysis cannot be performed at present as only young male mice data is available. Finally, our assumption/conclusions based on the result of bioinformatic analysis needs to be verified by experiment in the future. For instance, although we concluded that brain-infiltrating T cells rather than microglia might be more influential in mediating sexual dimorphism in stroke evidenced by those more striking changes of X chromosome genes between the sexes was observed in the T cells, their pathological functional could not be well addressed without experiment *in vivo*.

## 5 Conclusion

Our study integrated three series of transcriptomic data to conduct gene expression profiling in young and aged populations after cerebral hypoxia. We conclude that (i) X chromosome-encoded DEGs might contribute to sex-based differences in stroke incidence and severity throughout the age spectrum. (ii) Compared to microglia, gene expression pattern changes, especially those encoded on the X chromosome, are much more pronounced in infiltrating T cells after ischemic stroke regardless aging and sex, suggesting that targeting circulating T cells might play more critical role in mediating stroke (Muzny et al., 2012). The expression profiles of X chromosome-encoded DEGs in response to stroke are age- and cell type-dependent. These DEGs might be potential targets for future investigations exploring the mechanisms causing differences in stroke and other diseases where men and women are affected differently. Continued research and a personalized interventions to stroke treatment will be essential for addressing the unique needs of male and female ischemic stroke patients.

## Data Availability

The datasets presented in this study can be found in online repositories. The names of the repository and repositories and accession number(s) can be found in the article/Supplementary Material.

## Abbreviations

CAM, CNS border-associated macrophages; CNS, Central nervous system; CPC, Choroid plexus epithelial cells; DC, Dendritic cells; DEGs, Differential expression genes; EPC, Ependymocytes; GO, Gene ontology; HP, Human Phenotype Ontology; HPA, Human Protein Atlas; HS, Hemorrhagic stroke; IS, Ischemic stroke; LYM, Lymphocytes; MG, Microglia; MT, Mitochondria; NEUT, Neutrophils; OLG, Oligodendrocytes; PC, Pericytes; REAC, Reactome; SMC, Vascular smooth muscle cells; TLR, Toll-like receptor; vEC, venous endothelial cells; XCI, X-chromosome inactivation.
