# Supplementary material for: Profiling X chromosome genes expression relevant to sex dimorphism in stroke: insights from transcriptomics landscape analysis
Source: Front Genet. 2025 Mar 21;16:1479270. doi: 10.3389/fgene.2025.1479270 (PMC11968720; doi:10.3389/fgene.2025.1479270)
Supplement: Supplementary file 3 [file Supplementaryfile1.docx]

**Profiling X Chromosome Gene Expression Relevant to Sex Dimorphism in Stroke: Insights from Transcriptomics Landscape Analysis**

**The names of the repository/repositories and accession number(s)**

https://zenodo.org/records/6977557 (Zenodo database);

https://www.ncbi.nlm.nih.gov/geo/ (GSE137482 and GSE174574).

**Table S1.** Stroke dataset information

| **Comparison** | **Species** | **Cell/tissue type** | **Time after Stroke** | **Figure codes** |
| --- | --- | --- | --- | --- |
| **Female vs. Male** | Human | Whole blood, monocytes, neutrophils | 0-24 h, 24-48 h, and >48 h after stroke | 2, S1 |
| **Aged vs. Young** | Mice (C57BL/6J) | Parietal cortex tissue | 3 days after MCAO | 3 |
| **Stroke vs. Sham** | Mice (C57BL/6J) | Parietal cortex tissue | 3 days after MCAO | 3 |
| **Female vs. Male** | Mice (C57BL/6J) | Whole brain tissue | 3 days after MCAO | 4 |
| **Female vs. Male** | Mice (C57BL/6J) | Microglia and T cells | 3 days after MCAO | 5 |
| **Aged vs. Young** | Mice (C57BL/6J) | Microglia and T cells | 3 days after MCAO | 6 |
| **Stroke vs. Sham** | Mice (C57BL/6J) | 17 principal mice brain clusters | 24 h after MCAO | 7, 8, S2 |

**Table S2.** Multivariate linear regressions showing associations between risk factors of stroke and the differential expression of EIF2S3^*^ in the whole blood of ischemic stroke patients.

| **Models** | **Variables** | **Estimate** | **95% CI** | **t value** | **P** |
| --- | --- | --- | --- | --- | --- |
| **Model 1** | Male | -1.618 | -2.66 ― -0.58 | -3.156 | 0.0033 |
| **Model 2** | Male | -1.42 | -2.79 ― -0.05 | -2.111 | 0.0429 |
|  | Age | 0.000 | -0.05 ― 0.05 | 0.003 | 0.9978 |
|  | Diabetes | -0.457 | -1.87 ― 0.95 | -0.662 | 0.5126 |
|  | Hypercholesterolemia | -0.221 | -1.44 ― 0.99 | -0.372 | 0.7123 |
|  | Hypertension | 0.452 | -1.42 ― 2.32 | -0.56 | 0.5792 |

* The level of EIF2S3 was visibly presented in Figure 2C.


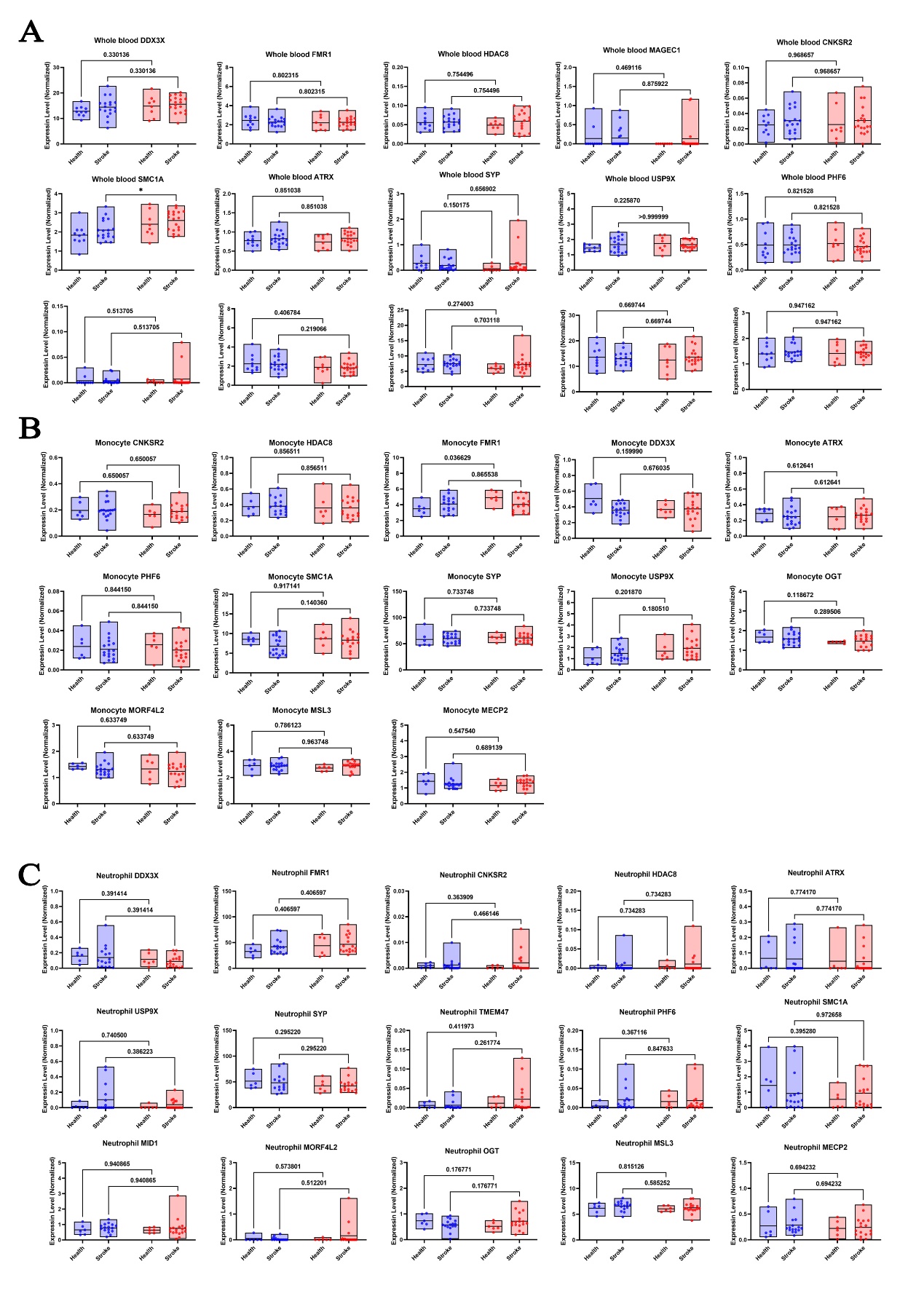


**Figure S1.** Sexual dimorphism of X chromosome escaping genes in response to ischemic stroke in human blood samples.

A. Expression profile analysis of ATRX, CNKSR2, DDX3X, FMR1, HDAC8, MECP2, MID1, MORF4L2, MSL3, MAGEC1, OGT, PHF6, SMC1A, SYP and USP9X genes in human whole blood samples. B. Expression profile analysis of the ATRX, CNKSR2, DDX3X, FMR1, HDAC8, MECP2, MORF4L2, MSL3, OGT, PHF6, SMC1A, SYP and USP9X genes in human monocyte samples. C. Expression profile analysis of the ATRX, CNKSR2, DDX3X, FMR1, HDAC8, MECP2, MID1, MORF4L2, MSL3, OGT, PHF6, SMC1A, SYP, TMEM47 and USP9X genes in human neutrophil samples (Data source: https://doi.org/10.1186/s12916-023-02766-1).


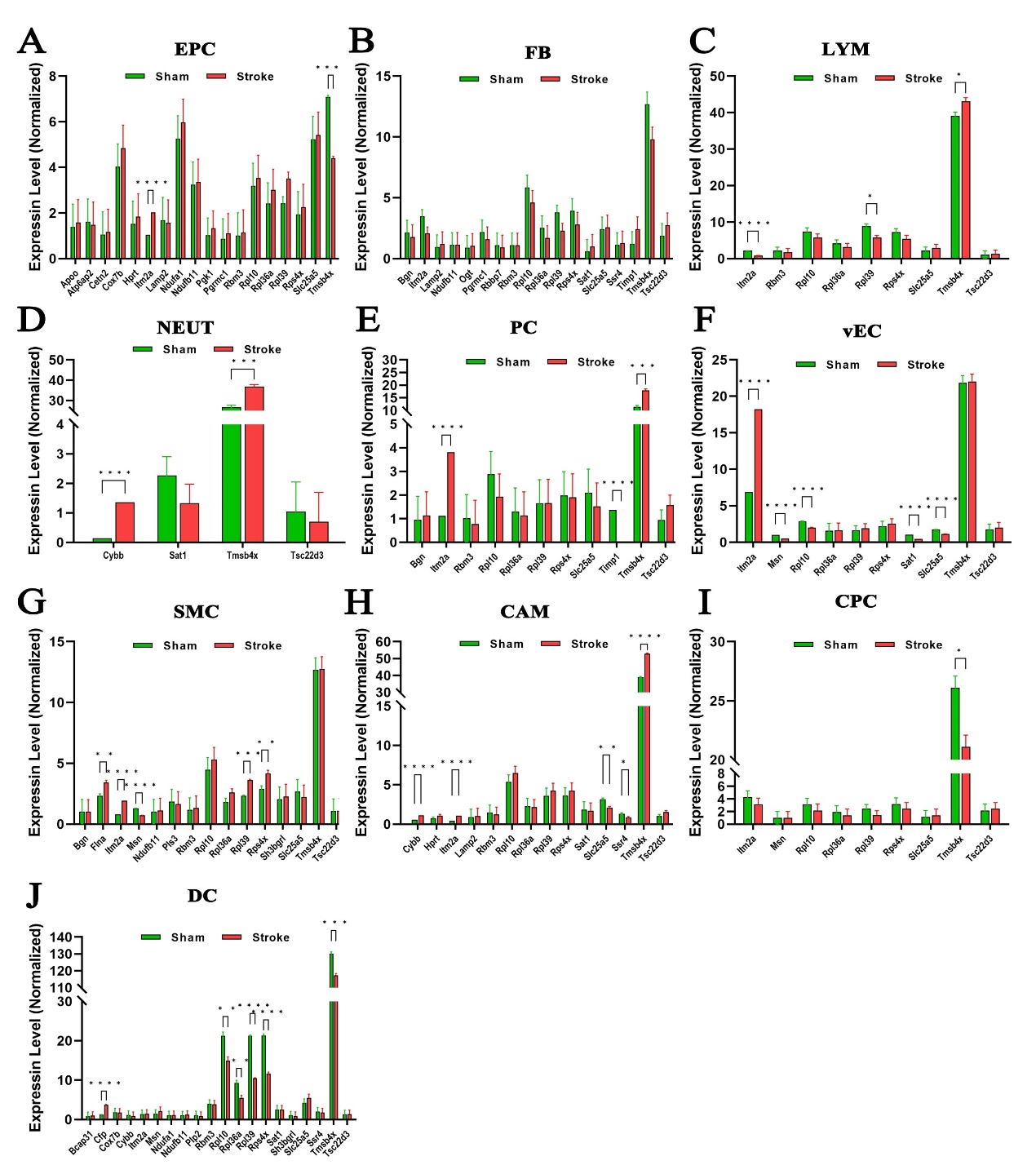


**Figure S2.** X chromosome gene expression profiles in different cell types of mouse brain.

X chromosome gene expression patterns of stroke vs. sham in ependymocytes (A), fibroblast-like cells (B), lymphocyte (C), neutrophils (D), pericytes (E), venous endothelial cells (F), vascular smooth muscle cells (G), CNS border-associated macrophages (H), choroid plexus epithelial cells (I), and dendritic cells (J) (Data source: GSE174574).


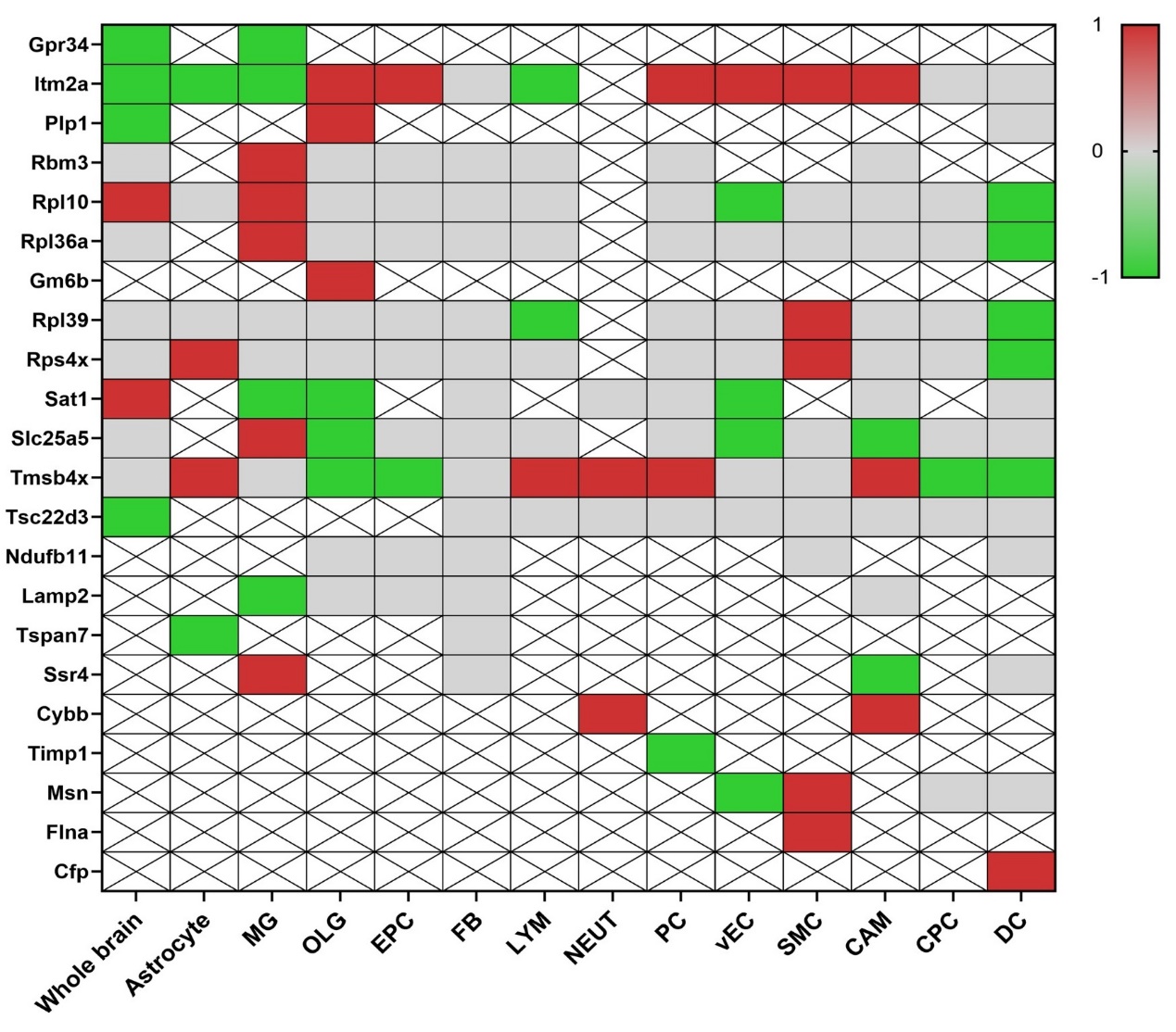


**Figure S3.** Heatmap showing the expression pattern of X chromosome-encoded genes in various brain cell populations (GSE174574).

Microglia (MG), oligodendrocytes (OLG), ependymocytes (EPC), perivascular fibroblast-like cells (FB), lymphocytes (LYM), neutrophils (NEUT), pericytes (PC), venous endothelial cells (vEC), vascular smooth muscle cells (SMC), CNS border-associated macrophages (CAM), choroid plexus epithelial cells (CPC), and dendritic cells (DC). 1 (Red cells): upregulation; -1 (Green cells): downregulation; 0 (Grey cells): no significant change; blank indicates data was unavailable. The list version can be found in table 2.
